# Multigenerational diabetes mellitus

**DOI:** 10.3389/fendo.2023.1245899

**Published:** 2024-01-15

**Authors:** Jennifer M. Thornton, Nishel M. Shah, Karen A. Lillycrop, Wei Cui, Mark R. Johnson, Natasha Singh

**Affiliations:** ^1^ Department of Academic Obstetrics & Gynaecology, Chelsea & Westminster NHS Foundation Trust, London, United Kingdom; ^2^ Department of Metabolism, Digestion & Reproduction, Faculty of Medicine, Imperial College London, London, United Kingdom; ^3^ Institute of Developmental Sciences, University of Southampton, Southampton General Hospital, Southampton, United Kingdom

**Keywords:** gestational diabetes, multigenerational diabetes, transgenerational diabetes, epigenetics, metabolomics, adipogenesis

## Abstract

Gestational diabetes (GDM) changes the maternal metabolic and uterine environment, thus increasing the risk of short- and long-term adverse outcomes for both mother and child. Children of mothers who have GDM during their pregnancy are more likely to develop Type 2 Diabetes (T2D), early-onset cardiovascular disease and GDM when they themselves become pregnant, perpetuating a multigenerational increased risk of metabolic disease. The negative effect of GDM is exacerbated by maternal obesity, which induces a greater derangement of fetal adipogenesis and growth. Multiple factors, including genetic, epigenetic and metabolic, which interact with lifestyle factors and the environment, are likely to contribute to the development of GDM. Genetic factors are particularly important, with 30% of women with GDM having at least one parent with T2D. Fetal epigenetic modifications occur in response to maternal GDM, and may mediate both multi- and transgenerational risk. Changes to the maternal metabolome in GDM are primarily related to fatty acid oxidation, inflammation and insulin resistance. These might be effective early biomarkers allowing the identification of women at risk of GDM prior to the development of hyperglycaemia. The impact of the intra-uterine environment on the developing fetus, “developmental programming”, has a multisystem effect, but its influence on adipogenesis is particularly important as it will determine baseline insulin sensitivity, and the response to future metabolic challenges. Identifying the critical window of metabolic development and developing effective interventions are key to our ability to improve population metabolic health.

## 1 Introduction

The hypothesis of the Developmental Origins of Health and Disease (DOHaD) is supported by evidence gathered over the past three decades, and describes how the environment that supports the developing fetus may affect the likelihood of disease development in adulthood (1–5). GDM, or diabetes occurring for the first time during pregnancy, is known to alter the uterine environment and increase the risk of short and long-term adverse outcomes for mother and offspring (6–8), including the risk of maternal Type 2 Diabetes (T2D) and offspring metabolic disease (6, 9). Up to 20% of offspring of GDM pregnancies develop T2D or pre-diabetes by the age of 22, and they are 29% more likely to develop early-onset cardiovascular disease (6). However, the mechanisms responsible and the period of pregnancy when they exert their effect are unknown, making it difficult to identify the optimal window of opportunity for health promotion in the preconception and intrauterine periods. Multiple interventions to reduce the risk of GDM and its negative effect on the developing fetus have been trialed. Lifestyle interventions in pregnancy have been shown to reduce the risk of GDM development in high-risk individuals, largely through reducing gestational weight gain, though effects are less pronounced in those at lower risk (10, 11) and have had little impact in reducing the fetal overgrowth seen in GDM (12). However, it is apparent that the earlier and the more intense the lifestyle intervention is, the more effective it is at reducing the risk of GDM (13), thereby highlighting the importance of early identification of high risk individuals (14).

Currently, the primary fetal phenotypic manifestation of exposure to an adverse intrauterine environment in GDM is the clinical detection of fetal overgrowth or macrosomia (large for gestational age, LGA), though somewhat counterintuitively, small for gestational age (SGA) infants are also observed. Both LGA and SGA infants are associated with poorer neonatal outcomes than infants that are average for gestational age (15). Excessive fetal growth in GDM is directly linked to maternal glycaemic control (7), which has also been demonstrated to increase birthweight and neonatal adiposity in a linear fashion in non-diabetic women (8). More recently, predictors of infant adiposity are thought to be closer related to maternal body mass index (BMI) and lipid, rather than glycaemic levels (9, 16), which are the current focus of GDM management protocols. SGA is linked to genetic factors and pre-eclampsia, however is also observed in strictly controlled GDM (15), particularly in women with lower pre-pregnancy BMI and lower fasting blood glucose levels (17). For this population, debate remains as to whether less stringent blood glucose targets should apply.

Achieving maternal blood glucose targets such as a fasting level <5mmol/l have been associated with reduction of many adverse outcomes in GDM pregnancies, but there is also now increasing evidence that glucose variability, rather than that related to specific timepoints, correlates with fetal outcomes (9, 18, 19). Variability in glycaemic control can trigger endothelial dysfunction and oxidative stress, leading to tissue damage, seen even in mild GDM (9, 20). Episodic hyperglycaemia may induce epigenetic changes, correlating with persistent diabetic complications (21). Interestingly, animal studies have shown that even when glycaemic control is achieved, complications from previous hyperglycaemia may progress via metabolic memory due to the production of excessive reactive oxygen species, advanced glycation end products and alterations in tissue-wide gene expression patterns (22, 23). Insulin resistance seen in GDM, especially hepatic insulin resistance, can exacerbate these processes by inducing pancreatic beta cells hyperplasia, which combined with hypertriglyceridaemia and hyperinsulinaemia, may precede the onset of global insulin resistance and T2D (24).

**Figure 1:**
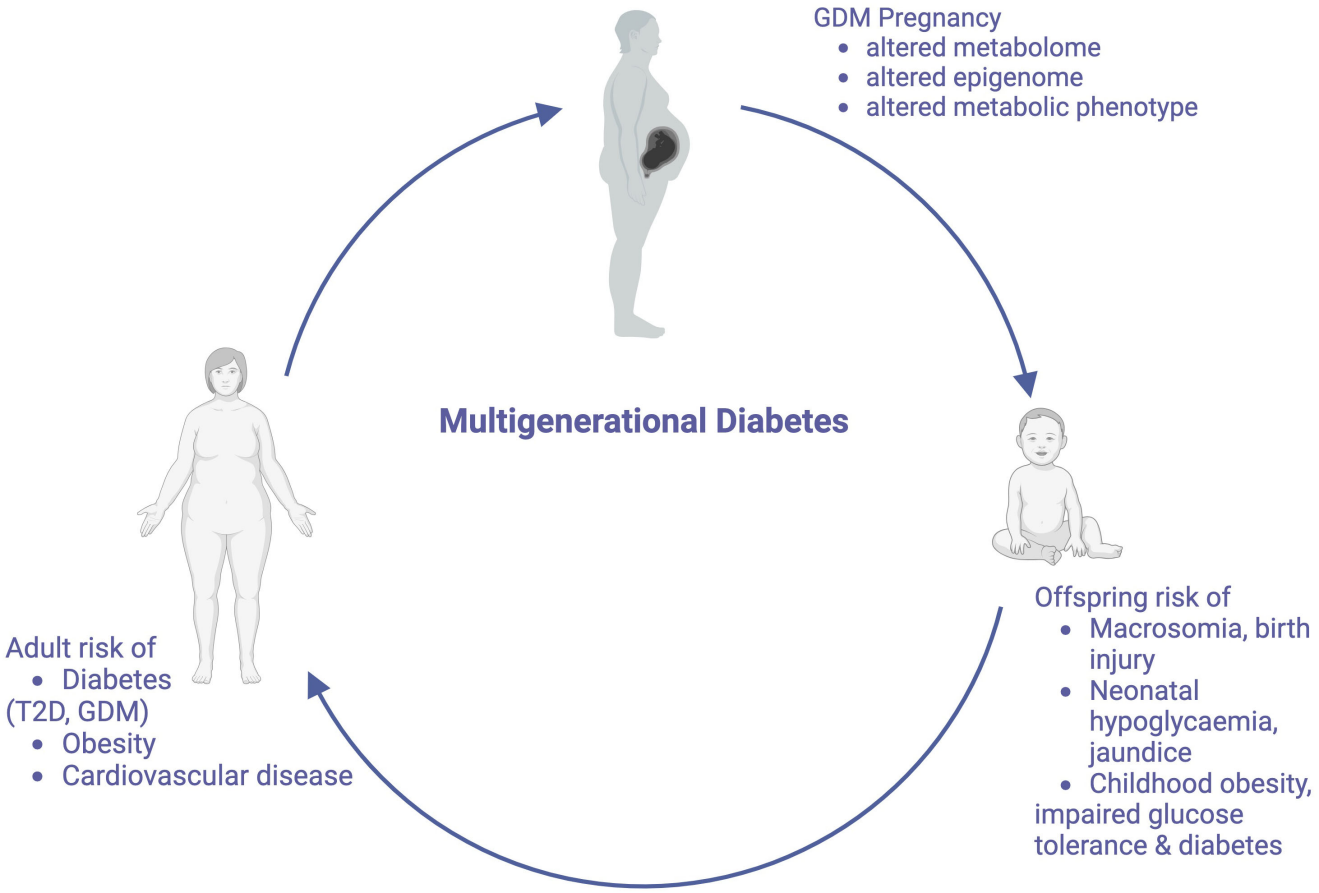
The Multigenerational Cycle of Diabetes. Pregnancies affected by GDM increase risks for mothers and their offspring, including the offspring’s future risk of developing GDM, thus perpetuating this cycle. Created with BioRender.com.

It is now well recognized that nutritional and environmental stimuli *in utero* precipitate changes to the offspring epigenome, and cause alterations in pathways associated with appetite regulation, metabolic signaling and energy storage (24–28). These stable modifications may explain how genome changes may be transmitted independently of changes to offspring DNA sequence. As these children become adults and subsequently have children themselves, this increased metabolic risk may also be propagated across generations (**Figure 1**). Distinction is made between the terms “multigenerational” and “transgenerational” disease. Multigenerational transmission refers to the direct influence of an environmental exposure on multiple generations, such as seen in F0 and F1 generations (29). Transgenerational inheritance requires germ line transmission, not involving direct environmental exposure, such as seen in epigenetic alterations to sperm, which become imprinted (29, 30). Transgenerational inheritance requires permanent alterations in DNA sequence of the epigenome. Both multi- and transgenerational transmission may contribute to the increasing the global burden of this diabetes. In this article, we review the literature to explore the mechanisms that underpin the multigenerational effects of GDM, however will also comment on transgenerational transmission, and the proposed therapeutic targets to break this cycle.

## 2 Subsections

### 2.1 GDM pathophysiology

Multiple maternal physiological adaptations occur in pregnancy to support the developing fetus. Changes in insulin sensitivity are designed to promote fetal nutrition. In the first trimester, maternal insulin sensitivity increases moderately, leading to increased lipid storage in maternal adipose tissue (AT) for later use during pregnancy and breastfeeding. Enhanced lipogenesis, adipocyte hyperplasia and increased AT lipoprotein lipase activity further increase maternal fat deposition as pregnancy progresses (31, 32). Obese pregnant women demonstrate higher glucose values than non-obese pregnant women, even in the absence of GDM (9), and higher pre-pregnancy BMI and early gestational weight gain (GWG) are modifiable risk factors, not only for GDM, but for adverse pregnancy outcomes in general (32).

**Figure 2:**
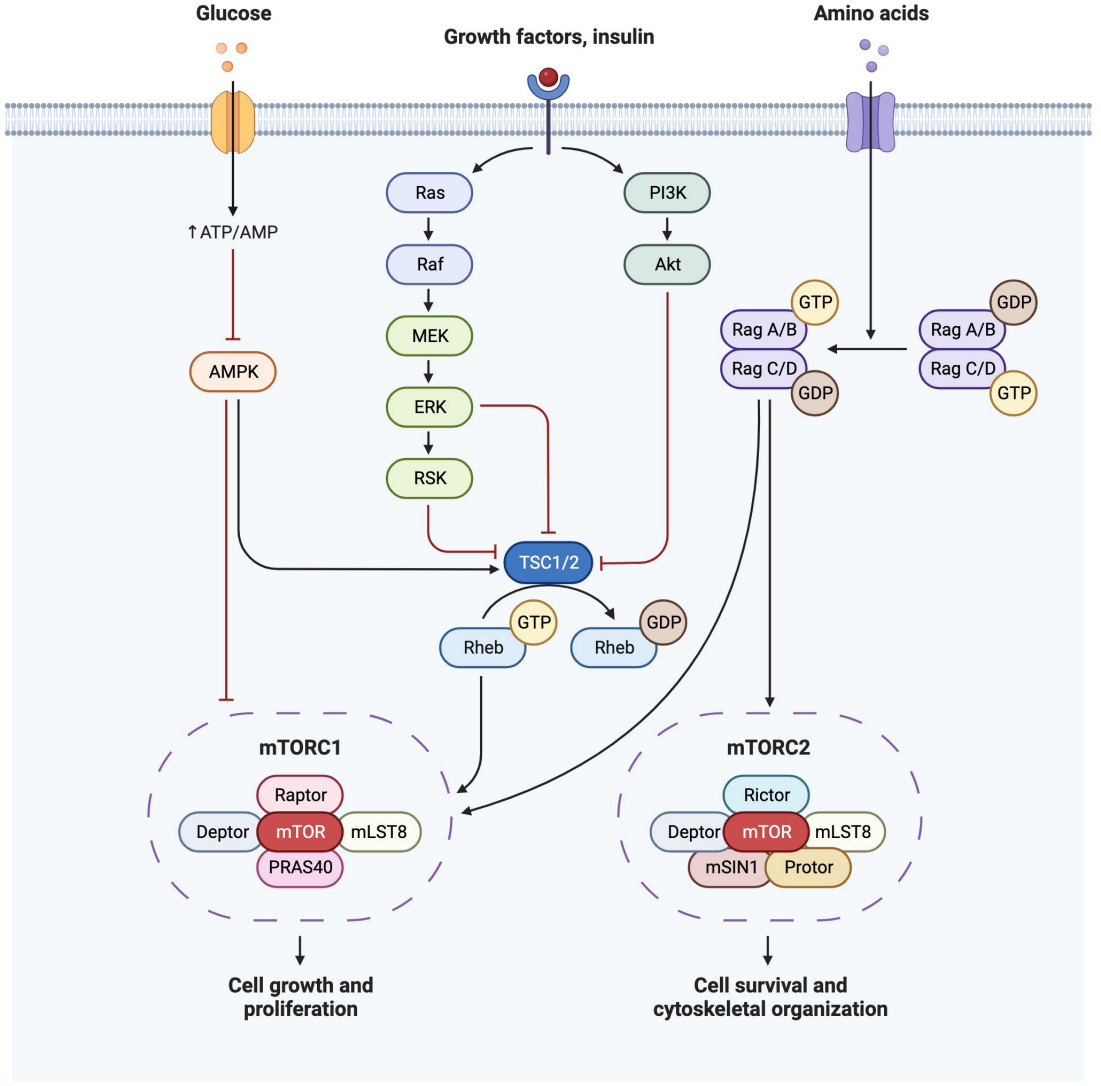
Insulin signaling pathway demonstrating PI3K/Akt pathway and MAPK(Ras/Raf) pathways and their downstream effects on mTOR. Created with BioRender.com.

Insulin sensitivity decreases by 50-60% with advancing gestation (33) and associated maternal hyperinsulinaemia increases the activity of placental pathways such as mTOR, and therefore nutrient transport to the fetus (6). Insulin is an anabolic hormone, which modulates glucose homeostasis by stimulating peripheral tissue glucose uptake, inhibiting hepatic glucose production, and suppressing AT lipid release (6, 34). In target tissues, insulin binds to the insulin receptor, allowing IRS-1 and IRS-2 to dock. Expression of the AT insulin receptor is decreased in the third trimester in GDM (32), with associated implications for glucose uptake, and for suppression of hepatic glucose output. There are two main insulin signaling pathways: the PI3K/Akt pathway (directs the primary metabolic function of insulin) and the MAPK pathway (regulates mitogenic effects of insulin) (34) (**Figure 2**). Increased IRS-1 serine phosphorylation is a major mechanism in insulin signaling inhibition (31) and is modulated by TNFα, which also reduces insulin receptor tyrosine kinase activity (33), thereby inhibiting insulin secretion and insulin-regulated glucose uptake. TNFα is a pro-inflammatory cytokine which rises during pregnancy and is also associated with insulin resistance outside of pregnancy in obesity, aging and sepsis (35). Insulin binding to subcutaneous adipocytes is reduced by more than 50% in the third trimester compared to outside of pregnancy, with further reductions seen in obese pregnant women (32). Insulin-mediated whole body glucose disposal further decreases by 50%, as decreased IRS-1 tyrosine phosphorylation causes a decrease in GLUT4 translocation to the cell surface, and in obese women there is also lower expression of GLUT1, thus affecting glucose transport (33).

Pregnancy-related insulin resistance becomes pathological (as in GDM) when maternal insulin production is unable to increase sufficiently to meet the pregnancy-induced changes in insulin sensitivity, resulting in maternal hyperglycaemia, and consequently increased fetal glucose levels. Women with GDM also have larger visceral adipocytes that correlate with plasma glucose levels (32). Chronic excessive calorie intake in pregnancy is linked to increased inflammation of the placenta and maternal AT. Inflamed AT is less sensitive to the antilipolytic effects of insulin, leading to increased hydrolysis of stored triglycerides to free fatty acids (FFAs) and providing further fetal nutrition. FFAs bind macrophage toll-like receptors, inducing NF-κB and perpetuating a cycle of inflammation and insulin resistance (31, 33, 34, 36). Higher basal and lower stimulated lipolysis are notably predictive of later development of insulin resistance, independently of BMI and age (37). Correlating with reduced maternal insulin sensitivity, and in response to hyperglycaemia, AT and the placenta secrete exosomes containing apolipoproteins implicated in the complement and coagulation cascade and cholesterol metabolism (33, 38).

In obese women, increased adiponectin, leptin, human placental lactogen, placental growth hormone and adipokines such as TNFα further exacerbate low grade inflammation and insulin resistance (31). Circulating leptin levels are attributable to maternal and fetal AT as well as the placenta, and increase to a maximum between 20 and 30 weeks of gestation. Leptin plays a role in regulating body fat mass, glycogen synthesis, fatty acid metabolism and modulation of insulin’s hepatic action (8, 39). The cycle of insulin resistance, AT inflammation and lipolysis accelerates in the third trimester, coincident with rapid fetal fat accretion and thus potentially macrosomia due to GDM-related fetal overnutrition (31).

#### 2.1.1 Placental transport

Maternal diabetes is associated with structural and functional placental changes, such as increased placental weight, delayed villous maturation and impaired mitochondrial function, occurring due to increased oxidative stress and alterations in the activity of amino acid transporter proteins (33). Maternal adiponectin has been linked to placental permeability (9) and is thought to promote insulin signaling and lipid oxidation and reduce gluconeogenesis (35), with decreased maternal levels noted in those giving birth to LGA infants (9). The majority of the reported changes in placental gene expression in women with GDM correlate with fetal adiposity and relate to lipid transport pathways, rather than glucose transport pathways, for example, hyperinsulinaemia upregulates placental *lipoprotein lipase* (*LPL*) (31, 40). Lipids activate cell signaling pathways and act as nuclear receptor ligands, therefore increases in lipid exposure *in utero* may lead to alterations in gene expression.

Increased placental expression of TNFα and IL-6 in GDM worsens local inflammation and insulin resistance (35). In turn, maternal insulin resistance also drives higher transplacental glucose transport via facilitated carrier-mediated diffusion (40). Subsequent fetal hyperglycaemia leads to fetal pancreatic beta cell hyperplasia, hypertrophy and insulin secretion, increasing glucose uptake and stimulating hepatic triglyceride and glycogen synthesis (9, 40).

mTOR is an important regulator of both glucose metabolism and lipogenesis, shown to be hyperactivated by over-feeding (41). mTOR is a serine/threonine protein kinase, and forms the catalytic subunit of two complexes (mTORC1 and mTORC2). mTORC1 regulates anabolism and catabolism and promotes protein synthesis through S6K1 and 4EBP phosphorylation. mTORC1 is also a promoter of *de novo* lipid synthesis through SREBP transcription factors, controlling gene expression of components of fatty acid and cholesterol biosynthesis pathways. mTORC1 activation further increases the synthesis and deposition of triacylglycerols (TG) in white adipose tissue (WAT) and stimulates PPARγ-driven pre-adipocyte differentiation (41). mTORC1 is reported to facilitate cell growth by shifting glucose metabolism from oxidative phosphorylation to glycolysis (42). Stimulation of the mTORC2-Akt axis by insulin induces glucose uptake and enhances storage as glycogen.

Overnutrition, leading to chronic hyperactivation of mTORC1 can disrupt PI3K-mTORC2 signaling, inducing insulin resistance, and ectopic lipid accumulation (41). mTORC1 dysregulation can impact insulin resistance in a sex-specific manner. For example, a mouse model of diabetes overexpressing 4EBP-1 only protects male mice from obesity and high fat diet-induced insulin resistance (34). Both human and animal models of GDM demonstrate alterations in placental mTOR signaling (43–45), phosphorylation of p70S6K, a downstream effector of mTOR, is increased in GDM (43). A rat model of multigenerational diabetes in pregnancy reported reduced placental expression of 4EBP-1, PKCα, PPARα and PPARγ and increased SGK1 phosphorylation, lipoperoxidation, nitric oxide production and peroxynitrite-induced damage in the first generation. This finding supports the theory of *in utero* metabolic programming leading to impairment of placental signaling pathways and increased pro-inflammatory uterine environment in subsequent generations (45).

Further to this, increased placental mTOR activity is associated with fetal overgrowth (44, 46). Placental mTOR and IGF-1 signaling pathways have therefore been proposed to contribute to macrosomia in GDM pregnancies (44). Therapies targeting hyperglycaemia in GDM may also impact placental nutrient transfer, and therefore fetal growth. Metformin, a commonly used oral hypoglycaemic agent, crosses the placenta and inhibits complex I of the mitochondrial electron transport chain, thereby downregulating mTOR cell growth and proliferation pathways. It has been demonstrated that metformin-exposed neonates are lighter than their insulin-exposed peers, independent of glycaemic control, though the long-term implications of this are as of yet unknown (47).

### 2.2 Metabolomic changes in GDM

Metabolomics refers to high-throughput analysis of metabolites and has been used in GDM in an effort to further understand changes in metabolic pathways, as well as to identify alternative diagnostic biomarkers. Significant heterogeneity exists between studies, including differences in sample type, timing of sample collection and sample size, making comparison between studies, and therefore establishment of meaningful conclusions, difficult. Diet, pharmacological treatment and environmental factors (48) also influence metabolite abundance, as well as genetic and epigenetic factors (49), further confounding comparability of findings. Metabolomic findings discussed in this review are summarized in **Table 1**.

**Table 1:** Metabolomic findings in GDM.

Sample Type	Sample Collection	Positive association with GDM development	Negative association with GDM development
Maternal fasting serum	First trimester	Short, saturated/low unsaturated TGs	Cholesterol esters, phosphatidylcholines, long polyunsaturated TGs, ceramides, sphingomyelins (50)
Maternal non-fasting serum	Second trimester	Short, saturated/low unsaturated TGs	Cholesterol esters, phosphatidylcholines, long polyunsaturated TGs, ceramides, sphingomyelins (50)
High-risk Patients -Maternal non-fasting serum	Second trimester	Phospholipid and free cholesterol in small HDL	Phospholipids in very large HDL, linoleic acid:total fatty acids, glutamine concentration (51)
Maternal fasting serum	Second trimester	Associated with GDM in all patients studied - Linoleic acid, glycoprotein acetyls, lactate - Total fatty acids, total MUFA, total SFA, and diameter of VLDL (stronger association in Western European patients) Associated with GDM in Western European patients only - Alanine, glutamine, total cholesterol, total n-6 PUFA, total PUFA and curate (49)	
Maternal fasting serum	Second trimester		Propionate (52)
Maternal fasting plasma	Second trimester	Galactitol, lactic acid and proline	Methylmalonic acid and glycerol (53)
Maternal plasma (not stated if fasting sample)	Second trimester	Glutamate, BCAAs (54)	
Maternal plasma (non-fasting)	Second trimester	Valine and pyruvate, betaine, lactate, fatty acids and triglycerides	Proline, urea, 1,5-anhydroglucitol, glutamine, creatine, dimethyl sulfone, trimethyl amine N-oxide (TMAO) (55)
Maternal Urine	Second trimester	Tryptophan and purine metabolism, hypoxanthine, xanthine, xanthosine, 1-methylhypoxanthine (56)	
Maternal plasma (non-fasting)	Third trimester	Betaine, alanine	TMAO, methanol and proline (55)
Maternal fasting plasma	Third trimester	Purine degradation metabolites (inosine monophosphate, hypoxanthine)	Medium-chain acylcarnitines (lauroyl-, octanoyl-, decanoyl-, decanoylcarnitine) (57)
High-risk Patients – Maternal fasting serum	Third trimester	Phospholipid and free cholesterol in small HDL, triglycerides in chylomicrons and extremely large VLDL, (MUFA and SFA):Total FA Fructosamine, glycolysis intermediate pyruvate, BCAAs (valine, leucine and isoleucine) and ACAAs (tyrosine) and acetoacetate	Total cholesterol, linoleic acid, omega-6, PUFA, adiponectin concentration (51).
Maternal fasting serum	Third trimester	BCAAs (leucine/isoleucine, valine), BCAA metabolites (AC C4/Ci4, AC C3, AC C5), Glutamate/glutamine (BCAA metabolic by-product), Aromatic acids phenylalanine and tyrosine, Gluconeogenic amino acids alanine, arginine, proline and asparagine/aspartate, Lipids (NEFA, glycerol, triglycerides, 3-hydroxybutyrate and its carnitine ester AC C4-OH), Lactate, Long-chain acylcarnitine, AC C16-OH/C14-DC Positive association between impaired glucose metabolism 10-14 years following GDM pregnancy and - Medium-chain acylcarnitines (AC C8, AC C10), Long-chain acylcarnitines AC C14:1, AC C14:2 and AC C16:1, AC C2 and 3-hydroxybutyrate and its carnitine ester AC C4-OH - leucine/isoleucine - NEFA - triglycerides, glycerol (58)	
Maternal fasting serum	Third trimester	Insulin resistance positively related to total VLDL lipids, total TG and TG : Phosphoglycerides, concentration of phospholipids in total VLDL, total FAs, SFAs, MUFAs, Alanine, valine and phenylalanine	Insulin resistance inversely related to total HDL lipids, total cholesterol concentration, concentration of phospholipids in total HDL, to proportion of linoleic acid, docosahexaenoic acid, omega-3 FAs and omega-6 FAs (59)
Maternal fasting serum	Third trimester		Butyric acid, acetic acid and total SCFAs (52)
Maternal Serum 1hour post glucose load	Third trimester	Medium- and long-chain acylcarnitines Positive association with impaired glucose metabolism 10-14 years following GDM pregnancy and leucine/isoleucine, valine, medium-chain acylcarnitines (AC C8, AC C10, AC C12), long-chain acylcarnitines (AC C14:1, AC C16:1, AC C2), 3-hydroxybutyrate and NEFA	Arachidonyl carnitine (AC C20:4) (58)
Maternal Serum 2hour post glucose load	Third trimester	Long-chain acylcarnitine, AC C16-OH/C14-DC (58)	-
Maternal Feces	Third trimester	Differences between GDM and non-GDM - hexadecanedioate, lysine, leucine, alanine, glycyl-leucine, putrescine, guanidinoacetate and isocaproate Enrichment of biotin metabolism in GDM (60)	
Maternal Feces	Third trimester	Microbiota altered in GDM, mainly Firmicutes bacteria genera, reduction alpha and beta diversity (53)	
Maternal Hair	Third trimester	Positive association between GDM, levels of air pollution and 2-hydroxybutyric acid, citramalic acid, myristic acid (48).	
Cell-culture based Progenitor cytotrophoblast and differentiated syncytiotrophoblast cells, exposed to NEFA	n/a	PA exposure associated with increased SFA levels (specifically C16:0) OA exposure increased cellular MUFA, particularly C18:1n9 (61)	-
GDM-exposed umbilical cord blood serum	Delivery	Alanine higher in infants of mothers treated with metformin, compared to those treated with insulin or lifestyle measures Smaller birthweight associated with large VLDL particle size, high TG:phosphoglycerides, elevated MUFA:total FA, increased DHA and increased omega-3 FA. Higher birthweight associated with Omega-6 FA (62).	-
Neonatal meconium	First-pass meconium	In neonates of mothers with GDM. - Significant reduction in alpha diversity - Relative abundance of Firmicutes and decreases in Proteobacteria - Increased abundance of riboflavin and taurine - Enrichment of carbohydrate and nucleotide metabolism pathways - Reduced glycerophospholipid, glycocholic acid and rhamnose (also seen in mothers’ serum) (63)	
GDM-exposed mice (pancreatic tissue)	Third trimester	Down-regulation of metabolites implicated in amino acid, lipid, carbohydrate and nucleotide metabolism. Most down-regulation of BCAAs (valine and leucine), more pronounced in male offspring (64).	-

n/a, not applicable.

Branched chain amino acids (BCAAs) are involved in fatty acid oxidation, mTOR, JMK and IRS1 pathways. Higher BCAA levels are implicated in obesity, insulin resistance, T2D and cardiovascular disease (37, 54, 71). Increased ketone bodies in GDM inhibit proteolysis and reduce BCAA and ketogenic acid oxidation in skeletal muscle (72). BCAAs are consistently associated with GDM, particularly leucine/isoleucine and valine, as well as several carnitine esters of BCAA metabolites, and the BCAA metabolic by-product glutamate/glutamine (49, 51, 54, 58, 60). Glycine has also been implicated in diabetes development and is an excitatory neurotransmitter of N-methyl-D-aspartate receptors in beta cells, which accelerates their dysfunction and hyperglycaemia-induced apoptosis (54). Amino acid dysregulation is also seen in animal models of multigenerational GDM in a sex-specific manner. In one study, BCAAs valine and leucine of male murine offspring of GDM pregnancies were most altered by a GDM intra-uterine environment, and lipid, carbohydrate and nucleotide metabolism were also dysregulated (64). Intra-uterine hyperglycaemia may also lower methionine, affecting availability of methyl donors with associated implications for offspring epigenetic modifications (64). Interestingly, restoration of glycaemic control has been shown the abundance of lactate and pyruvate but otherwise has no major impact on the maternal metabolomic profile (55).

A study comparing metabolites in Western European and South Asian women with GDM reported that while linoleic acid, glycoprotein acetyls and lactate were associated with GDM in women of both ethnicities, many of the other commonly reported GDM-associated metabolites were only found in Western European women (49). This highlights the influence of genetics on the metabolome, the potential heterogeneity in disease pathways between populations, and therefore the importance of carrying out metabolomic research across diverse populations.

Pre-pregnancy adverse lipid profile may have a future role in predicting GDM, as early second trimester lipid biomarkers have been shown to correlate with later oral glucose tolerance test (OGTT) (9). FFAs, phosphatidylcholines (PCs) and lysophosphatidylcholines have strong positive relationships with the risk of GDM development (72), however their utility as diagnostic markers wanes as pregnancy progresses (50). Interestingly, secondary analysis of high-risk women from the UPBEAT trial reported consistent association of both second and third trimester phospholipids and free cholesterol in small HDL with GDM (51), highlighting their potential use as biomarkers, particularly in high-risk individuals. Maternal serum triglyceride levels are notably the strongest predictor of macrosomia and are positively related to insulin resistance (59). Palmitate (PA) and oleate (OA) are the most abundant circulating NEFA (non-esterified fatty acids) in the serum of pregnant women, and are increased in GDM, which may contribute to altered placental lipid levels (61). Lipids, in particular PCs, may disrupt glucoregulatory mechanisms, thereby preceding hyperglycaemia. Dietary fats are thought to influence cytokine expression, inflammation and insulin resistance, and fetal exposure can activate proinflammatory pathways, which may then affect substrate metabolism, mitochondrial function and stem cell fate (31). Excessive fatty acid oxidation, depleted TCA intermediates and insulin resistance may cause overload of maternal mitochondria. This leads to incomplete fatty acid oxidation and an increase in plasma acylcarnitines, though acylcarnitines are noted to decrease as pregnancy progresses, possibly due to dietary changes (57).

Tryptophan and purine metabolism have been found to be directly associated with GDM progression (56). The kynurenine pathway, which produces nicotinamine adenine dinucleotide (NAD+) from tryptophan, is thought to be activated in GDM before placental hormones can produce a physiological effect (56). Increased levels of purine degradation metabolites (inosine monophosphate and hypoxanthine) are also seen in GDM, T1D and T2D (57). Uric acid is a downstream product of hypoxanthine oxidation which is commonly seen in diabetes, attributed to be due to concurrent generation of superoxide anions associated with increased inflammation and impaired insulin secretion (57).

Metabolomics has also been used in an effort to identify those that may go on to develop disorders of glucose metabolism, such as T2D, following a GDM pregnancy. GDM-related dysregulation of gut microbiota may lead to increases in TMA, TMAO, dimethyl sulfone and methanol, which may exacerbate low grade inflammation and contribute to T2D progression (55). Reduced sphingolipid biosynthesis and short-chain acylcarnitines negatively impact beta cell function and are associated with progression of GDM to T2D (72). Multiple metabolites detected during a GDM pregnancy are positively associated with the development of future disorders of glucose metabolism, such as medium- and long-chain acylcarnitines, leucine/isoleucine, NEFA and triglycerides (58). Identifying high risk individuals could help to focus diabetes prevention strategies and thereby increase their effectiveness.

Relationships have been identified between maternal metabolome in GDM pregnancies, and the metabolome of the neonate (53). Findings suggest that GDM may be associated with altered neonatal lipid and carbohydrate metabolism (63). In particular, the metabolites lysine, putrescine, guanidinoacetate and hexadecanedioate may be responsible for the separation of mother-matched neonatal metabolomes and neonatal changes may be driven by maternal hyperglycaemia-induced enrichment of maternal biotin metabolism (60). Caution in interpretation is needed however, due to the concurrent impact of mode of delivery and method of feeding on neonatal samples (63).

### 2.3 Metabolic phenotype – the role of glucose in adipogenesis and future metabolic health

#### 2.3.1 Adipogenesis as a marker of metabolic programming

AT may be classified into anabolic WAT and catabolic brown adipose tissue (BAT). AT buffers variation in energy supply and demand by integrating endocrine and metabolic responses (73). The capacity for this is exceeded during chronic overnutrition leading to pathological ectopic lipid accumulation, impairing insulin action in peripheral tissues and pancreatic insulin production (32, 73). Adipocytes play a role in systemic insulin sensitivity through secretion of bioactive lipid products such as palmitoleate, an independent determinant of insulin sensitivity (37).

Fatty acid esters of hydroxy fatty acids (FAHFAs) are decreased in serum and adipocytes in insulin resistant states and are mediated by ChREBP. FAHFAs promote insulin secretion, increase adipose glucose uptake and inhibit WAT inflammation and hepatic glucose production (37). Fatty acid-binding protein FABP4 is secreted following lipolysis stimulation, activating gluconeogenesis and stimulating hepatic glucose production (37). Pre-adipocytes have important immune, pro-inflammatory and haemostatic functions, and replicate in response to IGF-1, at a rate dependent on the depot from which they arise (74). IGF-1 driven preadipocyte utilization may contribute to greater loss of subcutaneous as opposed to visceral fat, thereby leading to metabolic dysfunction.

The primary role of WAT is energy storage (in the form of TG) and energy mobilization (in the form of FAs) (75). The average adipocyte size fluctuates with changes in body weight, and the site of hypertrophy has implications for the effect this may have. Visceral WAT hypertrophy is associated with hyperlipidaemia, while subcutaneous WAT hypertrophy is linked to insulin resistance (32, 75). Even healthy individuals with a family history of T2D demonstrate higher subcutaneous AT diameter compared to BMI-matched controls (32). The sympathetic system also controls fat cell number through inhibition of adipocyte proliferation (76). High output of fatty acids in visceral adipocyte hypertrophy also has direct effects on hepatic metabolism and is associated with M1-like macrophage mediated inflammation which may be implicated in insulin resistance (37, 75).

Fatty acid oxidation occurs to a much greater extent in thermogenic BAT and beige adipose tissue (BeAT). *PRDM16* stimulates brown adipogenesis by binding PPARγ, and is partly mediated by *PGC-1α* and *PGC-1β* (77). Adipocytes induced by *PRDM16* expression demonstrate higher levels of ELOVL6 and UCP1. ELOVL6 is a fatty acid, which in combination with stearoyl desaturase, promotes an increase in phospholipid oleic acid content, increasing plasma membrane fluidity and insulin signaling. ELOVL6 is encoded by *MLXIPL* and is the main transcriptional target of ChREBP in adipocytes (75). Changes in blood glucose and triglycerides associated with obesity are offset in individuals with BAT, and HDL levels are higher across all BMI categories, suggesting that BAT may mitigate the harmful effects of obesity. Active BAT in adults correlates with lower BMI, colder outdoor temperatures, female sex and, lower risk of cardiovascular disease and diabetes (78). Increased BeAT has also been associated with resistance to weight gain, even after HFD consumption in animal models (37).

In women with GDM, AT displays increased expression of inflammatory proteins and differential expression of proteins involved in mitochondrial dysfunction, sirtuin signaling and oxidative phosphorylation compared to BMI-matched controls (32). Visceral AT in women with GDM demonstrates higher macrophage infiltration, which correlates with insulin resistance, as well as higher expression and secretion of angiogenic proteins such as sflt-1 (32). Concurrently, hypertriglyceridaemia of pregnancy is exacerbated by GDM and pre-existing T2D (79).

#### 2.3.2 Offspring metabolic programming

Prolonged exposure to hyperglycaemia induces hyperinsulinemia in the fetus, leading to increased lipogenesis and fat storage in a sex-dependent manner (80). Prevalence of children becoming overweight and obese during their first decade increases significantly with maternal glucose levels and gestational weight gain. Furthermore, maternal insulin sensitivity is prospectively associated with neonatal fat mass with persistence of this relationship up to the age of 11 (40). Maternal macronutrient intake has been shown to correlate well with offspring macronutrient intake at 10y, with the strongest correlation seen between maternal prenatal diet and fat intake (31). Even in normal birth weight children, maternal GDM increased prevalence of becoming overweight/obese in first decade of life by at least 30% (81).

This suggests metabolic imprinting occurs with maternal hyperglycaemia even in normal birthweight offspring. Gestational nutrition promotes long-term fetal programming of the set point of an individual’s body weight. Dysnutrition can therefore determine physiological responses and lead to a permanent impaired energy balance later in life. Maternal obesity and diabetes is associated with high birth weight, which in combination with excessive neonatal nutrition and rapid catch-up growth, predisposes the offspring to fat accumulation (76).

In low risk pregnancies, maternal insulin resistance in mid and late pregnancy is significantly associated with fetal adiposity, but not fetal weight (40). There is a wide variation in antenatal fat mass and it is thought that antenatal adipose tissue accrual influences the susceptibility of offspring to postnatal obesogenic factors (40). In a murine model, maternal overfeeding is associated with higher fetal WAT mass, with enhanced PPARγ mRNA expression levels sensitizing to postnatal adiposity, which is more pronounced in female offspring (76). In humans, AT development is initiated during the 2^nd^ trimester, subcutaneous depots develop before visceral, and BAT develops before WAT (32). Maternal overnutrition is associated with increased NFκB activation, decreased AMPK signaling and increased PPARγ expression. AMPK signaling activates lipid oxidation, while PPARγ is the master regulator for differentiation of committed progenitors into adipocytes in all AT depots (31). Early PPARγ activation could also promote lipid storage in place of oxidative pathways, increasing the risk of obesity development. Adipocyte commitment occurs prenatally and the progenitors undergo dramatic postnatal expansion (40, 73). Adipocyte turnover is independent of BMI and adipocyte number is determined at an early age (8). Once attained, adipocyte number cannot be changed, highlighting the importance of targeted interventions during this window of development.

### 2.4 Genetics

Diabetes results from the interplay of multiple genes with environmental factors. Like T2D, GDM risk is partially attributed to variants of multiple genes, with varying effect across different ethnicities and nutritional environments. However, worldwide the different ways in which GDM is diagnosed and recorded, and the different timepoints and sample types tested affect the generalizability of study findings. Evidence also exists that GDM represents a heterogenous spectrum of disease. Lean patients with GDM demonstrate a secretory defect (30% of GDM), while obese patients demonstrate insulin resistance with hyperinsulinaemia and decreased insulin receptor binding (51% of GDM). Women with predominant insulin resistance are at higher risk of macrosomia and GDM-associated adverse outcomes, independent of BMI (82). In ten years following a GDM pregnancy, women still have up to a 60% increased risk of developing T2D and women with GDM have a 30% probability of having at least one parent with T2D (83), supporting the concept of multigenerational diabetes (14, 84). Unsurprisingly, strong associations exist between T2D-associated gene polymorphisms and GDM. The most identified genes in GDM are *TCFL7L2*, *MTNR1B*, *CDKAL1*, *KCNQ1* and *IRS1* (6, 85).

*TCF7L2* is a transcription factor involved in the Wnt-signaling pathway that has been suggested to be involved in almost 20% of T2D cases (86), and to increase risk of developing GDM by up to 1.9-fold (87) as well as to increase propensity toward weight gain (86). The genetic variant of *TCF7L2* conferring the highest risk of T2D is the single-nucleotide polymorphism (SNP) rs7903146 (88). This gene coordinates proinsulin expression and its processing into insulin, as well as adipogenesis and peripheral and hepatic insulin resistance (86, 88, 89).

The *CDKAL1* (CDK5 regulatory subunit associated protein 1 like 1) gene is highly expressed in the pancreas and in skeletal muscle, it inhibits cyclin-dependent kinase 5 (*CDK5*) and variants in *CDKAL1* can reduce insulin response to glucose intake (85, 90). Multiple SNPs have been linked to GDM, particularly in Asian populations (90), in particular, SNPs rs9295478 and rs6935599 increase GDM risk up to 2-fold (91).

The *MTNR1B* gene encodes a melatonin receptor, playing an important role in biorhythm regulation. In White European populations the *MTNR1B* SNP rs10830963 confers an approximately 10% higher risk of developing T2D (92) and up to a 26% higher risk of developing GDM (93), however the association has not been confirmed across different populations (92, 93). Melatonin inhibits insulin secretion through increased pancreatic melatonin receptor expression. Similarly, melatonin administration impairs glucose tolerance, particularly when administered in the morning (94).

SNPs in the *KCNQ1* (potassium voltage-gated channel subfamily Q member 1) gene have been reported to increase the risk of T2D development across multiple populations (95). This gene regulates insulin secretion through control of membrane depolarization of the pancreatic β-cells. The SNP rs2237895 has been found to confer a two-fold risk of GDM development (96), in particular, maternal allele transmission shows a higher associated risk of T2D compared to paternal transmission (97).

*IRS1* (Insulin receptor substrate 1) plays an important role in the insulin signaling pathway, as well as cell survival and growth. The SNP rs1801278 has been associated with both T2D and GDM, increasing the risk of GDM up to 3-fold (98).

GDM has also been linked to genes associated with maturity-onset diabetes (MODY), such as glucokinase (GCK, *MODY2* gene) which is implicated in glucose signaling, glycogenesis and insulin secretion (85).

Genetic variation in multiple metabolic pathways are demonstrated to influence GDM risk. CYP2E1 (cytochrome P450 2E1) is an abundant hepatic enzyme which participates in arachidonic acid metabolism. SNPs in the gene coding for CYP2E1, including C-1054T, are thought to affect lipid metabolism and insulin resistance, and have been was associated with GDM risk (27, 99). Concurrently, metabolites of arachidonic acid produced by CYP450 enzymes, known as epoxyeicosatrienoic acids (EETs), are proposed to enhance insulin sensitivity and glucose metabolism. EETs are hydrolysed to less active forms by soluble epoxide hydrolase (sEH) encoded by *EPHX2* (100). The predominant epoxygenases are CYP2J2, CYP2C8 and CYP2C9. The SNPs CYP2J2-rs76271683 and CYP2C8-rs11572177 have been associated with an increased risk of GDM, while the missense variant rs57699806 in *EPHX2* has been associated with an increased risk of GDM and higher 1hPG and G_AUC_ at OGTT (100). If multiple risk alleles are present, the risk of GDM may increase up to 1.8-fold (100).

### 2.5 Epigenetics

#### 2.5.1 Epigenetic mechanisms

Genetic profile is influenced by environment both *in utero* and postnatally, potentially resulting in epigenetic changes with implications for later development of disease, and transmission of disease risk to subsequent generations (if occurring during gametogenesis) (101). Epigenetic changes, i.e. the passage of genome modifications to daughter cells independently of changes in the DNA sequence, affect transcription factor accessibility and thus may be implicated in numerous different signaling pathways. The impact of an epigenetic modification is dependent on its position and on metabolic and hormonal stimuli (24). Modifications include DNA methylation (DNAm), post-translational modification of histone proteins and non-coding RNA regulation of expression, e.g. miRNA (31, 102).

DNA methylation is the most extensively studied epigenetic modification and is usually associated with transcriptional repression (24) as methylated CpGs are recognized and bound by methyl CpG binding proteins. These proteins block transcription factor binding or recruit transcriptional co-repressors or histone-modifying complexes. This promotes heterochromatin formation and induces transcriptional silencing. Upon fertilization there is global demethylation when the DNA methylation marks on the DNA of sperm and oocyte are largely removed with the exception of imprinted genes, then as differentiation proceeds there is a gradual increase in DNA methylation which creates tissue specific patterns of gene methylation and expression essentially creating distinct cell types (31). There is now substantial evidence from both experimental models and human epidemiological studies to suggest that the *in utero* environment can alter the epigenome of the infant leading to long term phenotypic changes; Maternal under-nutrition (103, 104), over-nutrition (105), micronutrient status (106) and maternal obesity (107) have all been associated with persistent changes in the methylation and activity of key metabolic genes in the offspring. A number of studies have looked to assess differences in DNA methylation patterns between mothers and offspring affected by GDM, however many of these studies have been small, used different definitions of GDM, different tissues (e.g. maternal blood, placenta, umbilical cord blood), and ethnically diverse populations (e.g. White European, South Asian) which coupled with different analytical methodologies utilized, make generalizability and comparison of studies more challenging.

#### 2.5.2 Maternal epigenetic modifications in GDM

Epigenome-wide association studies (EWAS) suggest that GDM affects maternal methylation and therefore also has an indirect impact on offspring methylation (108). Canouil et al. compared maternal blood and umbilical cord blood methylation and demonstrated high association between *TFCP2* gene methylation at cg22790973 and GDM exposure for both mother and offspring. GDM-exposed offspring methylation increased at the same rate as maternal methylation, compared to decreased methylation seen in non-GDM offspring (108). The function of this gene in different tissues is uncertain, however it is proposed to play a role in reproduction and embryonic development. Similar trends were seen at cg03456133, cg19107263 (located in *DLGAP2* gene body, most strongly expressed in the brain, and identified to have a role in diabetes) (97, 108), cg23355087, cg20002843 (within *LOC12784* gene, role uncertain) and at cg24440931 (within *H3C6*, functions in chromatin compaction) (108). Methylation at cg17065901 (within *FAM13A* gene, highly expressed in adipose tissue and identified to have a role in diabetes) increased in GDM offspring as maternal methylation increased, compared to non-GDM offspring. Methylation decreased at cg11493553 (within *UBE3C* gene, involved in protein polyubiquitination) in GDM-exposed offspring as maternal methylation increased (108). Other studies also add to the emerging body of evidence that maternal methylation may be involved in mediating effects on insulin sensitivity in future offspring (97, 108).

Fragoso-Bargas et al. reported an inverse association between insulin resistance and maternal blood hypomethylation at *TXNIP* CpGs (cg26974062, cg02988288, cg19693031), and CpGs at *SLC7A11* (cg06690548) and *ZSCAN26* (cg04861640) (109). They also described that though genetic variants across populations may have different effects, those associated with complex traits in more than one ancestry demonstrate consistent direction of effects (109). *TXNIP* indirectly encodes the GLUT1 receptor in the presence of glucose (110) and its expression is mediated by activation of *ChREBP* in pancreatic β-cells, regulating insulin production, hepatic glucose synthesis and peripheral glucose uptake. *TXNIP* is also highly expressed in AT and is upregulated in prediabetes (111). *TXNIP* is the most replicated DNA methylation marker associated with T2D, indeed alterations at *TXNIP* may also occur in response to obesity or hyperlipidaemia prior to the onset of T2D (112). *SLC7A11* expression is associated with higher insulin sensitivity in muscle and insulin resistance in adipose tissue. Hypomethylation of cg06690548 in *SLC7A11* has also been associated with high fasting insulin levels in nonpregnant populations (109). *SLC7A11* encodes a cysteine/glutamate plasma membrane transporter important for the formation of the antioxidant glutathione, which is thought to be deficient in T2D (109). Hypomethylation at this CpG is also thought to be related to BMI and blood pressure readings, and interacts with rs7006759 in *PSD3*, which is a gene with T2D-associated variants (109). *ZSCAN26* expression is associated with higher insulin sensitivity in both muscle and adipose tissue, and hypomethylation is seen in patients with T1D nephropathy, and is also associated with T2D risk (109).

MicroRNAs (miRNAs) influence mRNA translation and therefore protein expression, and so have been proposed as potential biomarkers for disease. Hromadnikova et al. examined first trimester maternal miRNA gene expression and reported upregulation of 11 cardiovascular disease-associated miRNAs in all the women who went on to develop GDM in their study. They reported on the potential utility of first trimester miRNAs as components of a predictive model for maternal GDM, particularly when clinical characteristics were also taken into account, however further research is needed on diverse populations before such an approach should be undertaken (113).

Lewis et al. performed a systematic review on maternal miRNAs in GDM however reported difficulty forming conclusions due to significant heterogeneity between studies (timepoints collected, diagnostic criteria used, inconsistent recording of ethnicity) and lack of adjustment for confounders (114). The most validated circulating miRNAs in maternal blood for GDM identified are summarized in the table below (**Table 2**). Target genes include *IRS1, IRS2, mTOR, PCK2* and *GLUT2*, all of which play a role in regulating glucose homeostasis (65, 69).

**Table 2:** Maternal blood miRNAs in GDM.

miRNA	Target	Regulator of
MiR-16-5p	*IRS1, IRS2, INSR, AKT1, AKT3, mTOR*	Insulin signaling pathway, pancreatic β cell proliferation, branched chain amino acids involved in insulin dysregulation (65)
MiR-17-5p	*Mitofusin1, mitofusin 2*	Cell proliferation and inflammation, vascular damage, mitochondrial function (66)
MiR-195-5p	*VEGFA* (placenta), *EZH2* (umbilical cells), *FADS2, ELOVL5, ACSL3, ACSL4, HADHA, CPT1A*	Cell proliferation, angiogenesis, lipid metabolism (67)
MiR-20a-5p	Unknown	Unknown, however linked to cardiovascular disease, T2D and fetal growth restriction (68)
MiR-210-3p	Unknown	Unknown, however linked to insulin resistance, hypoxia, anti-angiogenesis (69)
MiR-222-3p	*MGMT, PPP2R2A, RECL*	Unknown, however linked to obesity, T2D (70)
MiR-29a-3p	*PCK2*	Glucose homeostasis (69)
MiR-342-3p	*GLUT2, SNAP25*	Insulin secretion, hypothalamic impairment, excessive food intake (69)

#### 2.5.3 Placental epigenetic modifications

Multiple groups have examined differences in the placental epigenome in GDM pregnancies. Many have examined the expression of imprinted genes, which are dependent on their parent-of-origin. For example, paternally-expressed imprinted genes are inclined to enhance fetal growth, while the opposite is true for maternally-expressed imprinted genes (115). Their role includes that of nutrition provision regulation, rather than modulation of nutrition required by the fetus. *IGF2* is a one of the most important genes for regulating this balance, and paternally transmitted *IGF2* SNP alleles (rs10770125, rs2585, rs7924316, rs6578987, rs680 and rs4320932) have been linked to increased maternal glucose concentrations and GDM. The rs10770125 allele has been reported to reach genome wide significance (116). Studies on placenta and umbilical cord blood have confirmed that *H19* is reciprocally imprinted with respect to *IGF2*, and regulates its imprinting and expression (117). *IGF2* is more highly expressed in placenta and cord blood of pregnancies affected by GDM, and interestingly, is also more highly expressed in macrosomic pregnancies with normal glucose tolerance (NGT). Similarly, *H19* expression is lowest in GDM macrosomia (117).

*MEST* (human mesoderm-specific transcript) is an imprinted gene which plays a role in placental development. *MEST* is hypomethylated in placenta and cord blood of human GDM pregnancies (118–120). In a mouse model, *MEST* hypomethylation is associated with triacylglycerol accumulation in the placenta, abnormal mitochondrial function and increased adipocyte size (121). *MEST* hypomethylation is also observed in morbidly obese adults compared to those with normal weight (120), further supporting the influence of the gestational environment on long-term metabolic outcomes. GDM is also associated with hypomethylation of *NR3C1* in human cord blood and placental samples. *NR3C1* is a transcription factor mediating metabolic, stress response and behavioral pathways (118).

Maternal insulin sensitivity is associated with placental DNA methylation at a number of CpGs, 12 of which are known to be imprinted in the placenta (maternally imprinted: *SPHKAP, CNYN6, KCNIP4, PODXL, DLAGAP2, KCNQ1, DSCAML1, GPC6* and *OCA2*; paternally imprinted: *H19/MIR675, MCF2L* and *LINC01056*) (97). Further to this, five GRS (genetic risk scores) demonstrated an association between lower Matsuda index and higher methylation levels at cg01618245 (*CHRNA4*), cg12673377 (*MICALL2/UNCX*), cg24475484 (*DLGAP2*), cg08099672 (*ENTPD2*) and cg03699074 (*BDP1P*) (97).

GDM-induced changes to placental DNA methylation, proportional to maternal glycaemic levels, have been reported at the *LEP* and *ADIPOQ* genes, implicated in appetite regulation and metabolism (120, 122). Maternal hyperglycaemia is also associated with placental DNA methylation of *PRDM16* and *PPARGC1A*. *PRDM16* is a key regulator of brown adipose tissue differentiation (97). It interacts with C-terminal binding protein 2 (*CTBP2*) transcriptional co-repressor to block induction of genes required for muscle cell or white adipocyte differentiation from myogenic factor 5-positive (MYF5+) or MYF5- (likely precursors of beige adipocytes) precursor adipocytes. Lower methylation of *PRDM16* is associated with higher fasting glucose levels in the second and third trimesters and also correlates with umbilical cord blood leptin levels and birthweight (123). *PPARGC1α* is required for mitochondrial biogenesis and thermogenesis induction through adrenergic activation in brown and beige adipocytes, and is involved in glucose and lipid metabolism. Higher DNA methylation of placental *PPARGC1α* is associated with higher second trimester fasting glucose levels and 2h OGTT levels, as well as higher cord blood glucose (123). The bone morphogenetic protein 7 (*BMP7*) induces expression of *PRDM16* and PPARGC1α genes and lower *BMP7* DNA methylation is associated with higher 2h OGTT levels (123).

Heterogeneity in study method and design results in variation in reporting of differentially expressed genes in GDM placentas. Lu et al. controlled for maternal BMI and identified 2779 differentially methylated regions (DMRs) spread across different chromosomes, many of which were located in promoter regions. They also identified 401 hyper-DMGs (differentially methylated genes) and 288 hypo-DMGs, demonstrating enrichment in neural and developmental, endocrine, immune-related and cognition related pathways. Association was reported between the hyper-DMGs and oxytocin, calcium and insulin signaling, while hypo-DMGs were associated with T2D (124). Tang et also reported enrichment in pathways correlating with glucose and lipid metabolism (125), while Yang et al. reported upregulation of the estrogen signaling pathway and antigen processing and presentation (126). GDM is associated with altered maternal immune responses, and a greater proportion of cytokine producing NK cells (CD56+/CD3-/CD45+) and cytotoxic NK cells (CD16+/CD3-/CD45+) cells have been reported in GDM placentas (126). Yang et al. reported absence of the normally expressed RPS19-C5AR1 ligand-receptor complex in GDM placentas, supporting the theory that dysfunctional ligand-receptor interactions may play a role in GDM development (126).

Ding et al. reported differential expression in 281 mRNAs and 32 miRNAs (127), including upregulation of five mRNAs (*TBL1X, NOTUM, FRMD4A, SLC16A2* and *CLDN19*) and one miRNA (miR-202-5p) and downregulation of three mRNAs (*CCL18, HTRA1, SLC39A6*) and 4 miRNAs (miR-138-5p, miR-210-5p, miR-3158-5p and miR-4732-3p). In particular, miR-138-5p expression was negatively correlated with placental weight. Its target gene *TBL1X* was positively correlated with placental weight and significantly upregulated in GDM placentas, suggesting its role in altered placental nutrient transfer (127). Zhang et al. reported significantly higher placental expression of miR-135a-5p in GDM. Placental expression of *SIRT1* was also increased in GDM*. SIRT1* is a key metabolic sensor in various metabolic tissues and is the main gene target of miR135a-5p, which also regulates the AMPK signaling pathway, involved in glucose homeostasis, lipid metabolism, protein synthesis, cell proliferation and survival (128).

#### 2.5.4 Offspring epigenetic modifications in GDM

EWAS consistently demonstrate alterations in methylation of genes involved in metabolic pathways in GDM compared to non-GDM offspring (25). Hjort et al. compared umbilical cord blood methylation between these groups and annotated 58 differentially methylated CpGs to 56 unique genes, two of which (cg09452568 and cg0092687) map to *ESM1* and two of which (cg19739596 and cg14328641) map to *MS4A3* (25). *ESM1* plays a role in endothelium-dependent pathological disorders. *MS4A3* is involved in immune signal pathways and is also hypomethylated in the adipose tissue of adults with T2D (25). These genes are also implicated in nutrient sensing in enteroendocrine cells and in the signaling pathways of *TREM1* (monocyte and neutrophil activation), IL-1 (a pro-inflammatory cytokine) and sphingosine-1-phosphate (regulator of embryogenesis, cardiogenesis and vascular development) (25). However, pre-pregnancy BMI confounded methylation differences in all but 13 CpGs in this study, highlighting the overwhelming influence of maternal obesity on epigenetic programming (25).

Antoun et al. reported altered cord blood methylation of another 242 CpG sites associated with maternal GDM, of which 7 remained significant after adjustment for maternal BMI. These modifications affected transcriptional regulation, cell signaling and division pathways. These include cg03566881 (located within *LGR6* gene) and cg16536918 (within *AVP* gene) (28). Differentially methylated regions (DMRs) within *ZMYND8* and *PLEKHB1* were common to GDM, fasting plasma glucose (FPG), 1 hour plasma glucose (1HPG) and 2 hour plasma glucose (2HPG). *ZMYND8* acts as a template for histone peptide recognition and contributes to cell proliferation, migration and DNA repair pathways. It interacts with *BRD2* (contains a DMR associated with 1HPG and 2HPG), which has been reported to be associated with maternal hyperglycaemia. *BRD2* is highly expressed in pancreatic beta cells, inhibiting cell mitosis and insulin transcription (28). Antoun et al. also reported sexual dimorphism in infants exposed to hyperglycaemia, finding no overlap between the dmCpGs associated with 1HPG in males and females. Disparity in dmCpGs associated with the different components of the OGTT was also noted, as was greater attenuation of the methylation signature linked to these values following the implementation of a lifestyle intervention. Interestingly, the 1HPG level, representing insulin secretion impairment, demonstrated the most extensive CpG associations following adjustment for maternal BMI. The top 2 dmCpGs reported were cg0896944 (within the intergenic region of chromosome 17) and cg08960443 (within *TMEM210*). Further to this, 26 DMRs were associated with GDM and 1HPG including *THEMIS2, PLCH1, AVP, SPON1* and *OXT*. The 1HPG level may therefore greater influence fetal epigenetic changes, which may be modified by lifestyle intervention (28).

Tobi et al. found no evidence for robust associations between maternal prenatal glucose and insulin levels and offspring cord blood DNA methylation. However, significant associations were identified between a higher AUC_gluc_ and hypomethylation at cg26974062 and cg02988288 (located within *TXNIP*) (26, 110, 111). High heterogeneity was found for these associations, hypothesized to be due to the effect of management of hyperglycaemia in the third trimester on cord blood DNA methylation, as was noted during a randomized lifestyle intervention trial during pregnancy (12). Juvinao-Quintero et al. looked at maternal HbA1c as a marker of dysglycaemia and that a 1% unit increase in maternal HbA1c was associated with 1.7% lower cord blood methylation at cg21645848, located on the cell proliferation upregulator of the *URGCP* gene (27). This effect remained significant when controlled for maternal BMI. *URGCP* overexpression correlates with upregulation of TNFα, which reduces insulin sensitivity by facilitating phosphorylation of the insulin receptor. Maternal TNFα levels in pregnancy are independently associated with higher maternal insulin resistance and in non-pregnant individuals TNFα overexpression is characteristic of T2D (27).

In overweight and obese women, early pregnancy HOMA-IR has been associated with cord blood hypomethylation at cg03158092 (near the *SYN3* gene, part of synapsin gene family involved in neurological pathways) and cg05985988 (near the *JARID2* gene, implicated in embryonic development and differentiation of pancreatic β-cells), whereas early pregnancy insulin sensitivity has been associated with cord blood hypermethylation at cg04976151 (on the *POLR2C* gene, involved in protein coding) (129). In this population later in pregnancy, higher insulin concentrations were also associated with cord blood hypomethylation at cg12082129 (within *PCSK7* gene, may act as a mediator of adipocyte differentiation) and maternal glucose load changes associated with cord blood hypomethylation at cg03403995 (near the *OCA2* gene, plays a role in tyrosine transport) (129).

GDM management methods, including maternal exercise, are also associated with cord blood epigenetic modifications. Metformin, which is a widely used treatment for GDM and T2D, has been shown to be associated with an increase in *SIRT1* activity (positively regulates insulin signaling) and a decrease in class II HDAC and HMT activity (24).

GDM-related fetal epigenetic changes may also be linked to neonatal outcomes. Su et al. report that *IGF2* DMR CpG sites are more hypomethylated in GDM offspring compared to NGT offspring, and in macrosomic offspring of NGT pregnancies compared to normal birth weight offspring of NGT pregnancies, while the reverse may be found for *H19* DMR CpG sites (117). Birth weight itself was found to be negatively associated with methylation of *IGF2* DMR CpG sites 10 and 12, and positively associated with methylation of *H19* DMR CpG sites 15 and 16. Negative associations were also found between methylation of *IGF2* DMR CpG site 6 and fasting plasma glucose (FPG) levels at OGTT. These finding suggested that changes to *IGF2/H19* expression may link GDM with risk of macrosomia (117). Further to this, Lu et al. reported alteration in *ZNF423* methylation and expression in the umbilical cord blood of infants of GDM pregnancies, which was directly proportional to adipocyte size. *ZNF423*’s gene product is known to promote transformation of non-adipocytes to adipocytes, and to inhibit subcutaneous adipogenesis, leading to adipocyte hypertrophy and inflammation, and subsequently obesity and insulin resistance (124). Houshmand-Oeregaard et al. reported on skeletal muscle miR-15a and miR-15b expression in adult offspring who had been exposed to maternal diabetes in fetal life. Positive association was noted between expresson of miRNAS and FPG, 2hPG and HbA1c. In particular miR-15b was suggested to contribute to development of future insulin resistance by downregulation of the insulin receptor and upregulation of *PI3KR1*, leading to impaired insulin signaling (130).

#### 2.5.5 Imprinted genes in animal studies

Maternal and paternal metabolic syndrome have both been demonstrated to cause epigenetic re-programming and insulin resistance in offspring (24). In animal studies, offspring of mothers fed a high-fat diet (HFD) demonstrate hyperglycaemia and insulin resistance and hepatic epigenetic modifications. These include hypermethylated and altered gene expression with changes in active promoter histone mark enrichment (H3K14ac and H3K9me3) (24). Reduced repressive and increased active histones marks were also observed in the *Pck1* gene, which encodes hypermethylation of the *Irs2* gene and a rate-limiting enzyme in gluconeogenesis (*PEPCK*). The *SIRT1* histone acetylation pathway is also affected by maternal HFD. *SIRT1* overexpression is observed to attenuate with effects of a maternal HFD and interestingly, offspring metabolic alterations are shown to persist even with the introduction of a normal diet (24).

Germline epigenetic changes inherited through meiosis may explain transgenerational transmission. The mouse model of GDM allows us to mechanistically examine the changes observed across generations, as well as the impact of imprinted genes. The first and second generations of a mouse GDM pregnancy (F1, F2) demonstrate impaired glucose tolerance (IGT), which more severely affects male offspring (101). Subsequent to this, F2 born from an F1 parent with IGT demonstrate earlier IGT and hyperinsulinaemia regardless of which parent had IGT, and increased birth weight through the paternal line (101). In F2 offspring, pancreatic islet *IGF2* expression is downregulated most in the maternal line, while *H19* is most downregulated in the paternal line. Furthermore, regardless of whether F1 mice had IGT, *IGF2* and *H19* were noted to be downregulated in sperm of F1males (101, 131), highlighting the effect of intrauterine hyperglycaemia on germ cell gene expression and risk transmission. Other mouse models of GDM have sought to confirm this finding, and also noted offspring sex-specific differences in response to maternal hyperglycaemia. While IGT was reported in both male and female offspring, only male offspring demonstrated increased birth weight, increased gluconeogenesis, increased fasting insulin and decreased insulin sensitivity (132). In contrast to findings in the pancreas, hepatic expression of *IGF2* and *H19* was significantly higher in male offspring, with hypermethylation reported at *IGF2*-DMR0 and *IGF2*-DMR1. *FOXO1* was also expressed more in male offspring (132). *FOXO1* and *ChREBP* (carbohydrate-responsive transcription factor) are activated by CBP (carbohydrate-responsive binding protein) and p300 and enhance lipogenesis in insulin resistant states (24). *FOXO1* is active during fasting and interacts with *PPARGC1α* to upregulate G6PC (glucose-6-phosphatase) and PCK1 (cytosolic phosphoenolpyruvate carboxykinase 1), inhibiting the Akt-induced insulin pathway.

The imprinted genes *Gtl2/Meg3* have also been implicated in transgenerational diabetes risk in the mouse model. *Gtl2/Meg3* is upregulated in the placentae of F1 and F2 offspring of GDM pregnancies (131). *Meg3* is predominantly maternally expressed and binds the PRC2 chromatin modification complex in murine embryonic stem cells, controlling expression of *Dlk1, TGFB1* and *IGF2*. *Dlk1* promotes activation of the insulin/*IGF1* signaling pathway and inhibition of adipogenesis and *TGFB1* contributes to mediation of pancreatic differentiation. *Dlk1* expression is downregulated in F2 offspring, while *IGF2* is downregulated in placentae of both F1 and F2 (131). *Meg3* overexpression inhibits *TGFB1*-stimulated cell proliferation and apoptosis, while hyperglycaemia stimulates *TGFB1* expression. Hyperglycaemia in a mouse pregnancy leads to hypermethylation of *Dlk1*-DMRs and hypomethylation of *Gtl2*-DMRs in F1 and F2. These findings were confirmed in microarray analysis, demonstrating downregulated *Dlk1* expression with predominantly paternal transmission, and upregulated *Gtl2* expression with predominantly maternal transmission in F1 and F2 (131).

## 3 Discussion

GDM is an important global public health issue, demonstrated by the immediate and long-term impact on maternal and offspring metabolic health. Exposure to GDM impacts multiple pathways, including lipid metabolism, organ development and developmental and endocrine disorders (25), which may then perpetuate the vicious cycle of metabolic disease.

The global standardized prevalence of GDM is 14%, ranging from 7.8% in Europe to 27.6% in the Middle East and North Africa (133). Prevalence ranges vary due to differing screening strategies, diagnostic criteria and baseline population risk. Studies have indicated that in some women metabolic derangements precede pregnancy, and as such pre-pregnancy screening may help to identify higher risk individuals, and therefore enable pre-conception intervention. In particular pre-pregnancy FBG, insulin and HOMA-IR noted to be independent pre-pregnancy predictors of GDM (134). Elevated first trimester FPG (>4.6 mmol/L) was found to increase likelihood if developing GDM 4-fold in a Chinese population, while elevated BMI (>23.5 kg/m2) carried a 1.14-fold higher risk. Combining these two values as a screening test in the first trimester significantly increased GDM prediction, however this was accompanied by a high rate of overdiagnosis, which has implications not only for women, but healthcare resource management (135). Metabolomics studies report changes in maternal metabolites from the first trimester in GDM pregnancies, however, biomarker discovery remains elusive due to the significant heterogeneity in studies, as well as the limited generalizability of metabolite findings across populations and different environmental exposures. Early second trimester lipid biomarkers have been suggested as a potential test to precede the OGTT, but large-scale cross-population direct comparison studies have yet to be undertaken.

Debate remains as to the optimum screening methodology for GDM, with both 1-step and 2-step glucose tolerance screening strategies in use, depending on the geographical location. Approximately twice as many women with GDM are identified using 1-step screening, including more women with milder hyperglycaemia (16), which has raised the issue of potential medical over-intervention. There is also debate as to whether targeted or universal screening should be implemented. Though risk factor-based screening is likely to select out high-risk individuals, most likely to experience GDM-related adverse events, it has been shown that such a strategy misses one third of GDM cases (136). Risk-factor based screening is only thought to be predictive for GDM-related events in European and North African populations (137). It should be remembered that even “mild” GDM is associated with rare but significant adverse perinatal outcomes when untreated, and that these can be avoided or reduced with management strategies (138, 139). For example, meta-analysis has demonstrated an 18% reduction in the risk of GDM with lifestyle intervention, with increased effect size (22%) when intervention was initiated earlier (before 15 weeks gestation) (140). Furthermore, pre-pregnancy adherence to Mediterranean diet also decreases the risk of developing GDM, regardless of weight and other risk factors, in particular, avoidance of pre-conception consumption of processed and fatty meats (141, 142). “Before the Beginning”, an RCT of preconception lifestyle intervention that is currently ongoing, is examining the effect of time-restricted eating and exercise on maternal glucose tolerance at 28 weeks gestation (143).Time-restricted eating has been demonstrated to be a feasible lifestyle intervention in those with obesity and T2D outside of pregnancy (144), however adherence in pregnancy may be more challenging outside of the more intensive support offered during an RCT.

Approximately 60% of women manage their GDM through lifestyle measures alone (145). However if this is not achieved, oral medication (such as metformin) and/or insulin is recommended. Maternal physical exercise stimulates glucose transporters onto the surface of skeletal myocytes, improving glucose uptake and therefore reducing insulin resistance. In overweight or obese women, initiating exercise early in pregnancy reduces the risk of GDM by 45% (33). Though pharmacological methods have a favorable safety profile in pregnancy, they are known to affect fetal growth. For example, Metformin-exposed neonates are lighter, have reduced lean mass and lower risk of macrosomia than insulin-exposed neonates, independent of maternal glycaemic control (47).

Multiple EWAS sought to examine the impact of maternal GDM on children, and therefore future population health. Significant heterogeneity also exists in this methodology, limiting the generalizability of findings. Variation in the tissue type studied, and its applicability to other tissues and future environmental exposures are a matter of debate. Treatment of maternal GDM as a binary variable, as opposed to examining the continuous relationship between maternal hyperglycaemia, other metabolic changes and offspring DNA methylation may also contribute to these differences. Difficulty in obtaining follow-up data has also been reported as a limiting factor. Luo et al. examined the relationship between prenatal GDM exposure and structural neurological measures in children up to 10 years of age. Importantly, this group included siblings discordant for GDM exposure in order to determine the effect of shared genetics and environment. They reported that GDM exposure was associated with lower global cortical grey matter volume and higher likelihood of childhood overweight/obesity. They suggest that the neurological changes observed may predispose children to poorer self-regulation, and therefore increased risk of obesity (146).

Prevention of progression to T2D and prevention of recurrent GDM, are two major focuses of current strategies to reduce metabolic impact on mothers and their children. In the first five years following a GDM pregnancy, up to 55% of women develop pre-diabetes, and 6% develop T2D (147). There is wide variation in reported GDM recurrence rates due to differences in diagnostic criteria and populations studied. In a systematic review, Kim et al. reported recurrence rates ranging from 30-84%, with higher rates of recurrence noted in non-white populations (148). BMI, interpregnancy weight gain, previous macrosomia and insulin treatment were reported by Schwartz et al. to be the main predictors of GDM recurrence (149). Phelan et al. recently published an RCT of pre-pregnancy lifestyle intervention to reduce rates of recurrent GDM (150). Unfortunately, though the lifestyle intervention was effective in producing weight loss, no significant effect on GDM recurrence was found. The authors reported a lower than anticipated pregnancy rate in their cohorts, which underpowered their study, and also discuss the effect of gestational weight regain in their intervention group. Evidently larger, multicentre RCTs are required to further assess the effect of this intervention. Most published studies focus on the progression of GDM to T2D, where intervention in women with impaired glucose tolerance is demonstrated to delay the onset of T2D (14). The Diabetes Prevention Program demonstrated that an intensive lifestyle intervention, involving dietary changes and moderate physical activity, halved the risk of developing T2D in women with previous GDM (151), however studies report difficulty with patient recruitment and adherence due in part to difficulties balancing intervention with early-years childcare, social supports and subsequent pregnancies (152, 153). Haschka et al. reported that in postpartum women, glucose tolerance fluctuated from year to year, rather than steadily declined (147), which may potentially provide further opportunity for diabetes prevention. Progression to T2D is associated with higher pre-pregnancy BMI, advanced maternal age, family history of diabetes and postpartum lifestyle factors (154). Furthermore, GDM affects the entire family, partners of women with GDM have a 33% higher diabetes incidence than partners of those without GDM (155), highlighting the importance of shared environment and a household-approach to health promotion. Interventions aimed at reducing GDM progression to T2D tend to be more effective when commenced between 6 weeks and 2 years postpartum, and when they last for a year or more (154, 155). Investment in social supports, consistency of health promotion messaging and opportunistic, as well as targeted, interventions may all improve uptake and effectiveness of diabetes prevention strategies.

Integration of clinical information, consistency of diagnostic methods and standardization of metabolomic and epigenetic data collection and processing are essential, such that more effective diagnostic biomarkers and management policies may be established for the improvement of the metabolic health of mothers and their children.

## 4 Conclusion

Generational susceptibility to metabolic disease is multifactorial, involving the interplay of genetic risk and environmental influence. Identification of and intervention at the critical window of metabolic development, and therefore prevention of epigenetic modification-induced changes, has the potential to impact not only offspring fat mass and baseline insulin sensitivity, but also the robustness of the offspring to future metabolic challenges, such as obesity, T2D or cardiovascular disorders (31, 77). In the face of increasing global rates of GDM and its association with obesity, the need for a simpler, more sensitive screening strategy, as well as an assessment of current management policies has never been more important, to allow us to successfully break the generational cycle of Diabetes.

## Author contributions

JMT performed the literature review and wrote the initial manuscript. NMS, KAL, WC, MRJ, and NS edited the manuscript. All authors contributed to the article and approved the submitted version.
